# Handheld Optical Coherence Tomography in a Young Infant With Albinism and Fovea Plana

**DOI:** 10.1097/WNO.0000000000001094

**Published:** 2020-09-11

**Authors:** Sohaib R. Rufai, Helena Lee, Irene Gottlob

**Affiliations:** The University of Leicester Ulverscroft Eye Unit (SRR, IG), Leicester Royal Infirmary, Leicester, United Kingdom; Department of Ophthalmology (SRR), Great Ormond Street Hospital NHS Foundation Trust, London, United Kingdom; and Clinical and Experimental Sciences (HL), Faculty of Medicine, University of Southampton, Southampton, United Kingdom.

## Abstract

We present handheld optical coherence tomography (OCT) diagnosis of Grade 4 foveal hypoplasia (fovea plana) in a 28-day-old infant with albinism. Grade 4 foveal hypoplasia is characterized by the absence of the foveal pit, absence of outer segment lengthening, and absence of outer nuclear layer widening. Binocular visual acuity at 58 months follow-up was 1.2 logarithm of the minimal angle of resolution (logMAR). We describe our handheld OCT acquisition protocol and compare the morphological features with a healthy, age-matched control subject.

In this article, we present optical coherence tomography (OCT) imaging obtained from a 28-day-old Caucasian male patient with albinism, referred to our clinic for workup of infantile nystagmus. On examination, the patient had pendular nystagmus, transillumination defects, and cutaneous hypopigmentation. The patient was unable to cooperate with preferential looking testing.

Figure 1A displays successful handheld OCT acquisition in this patient, without dilation or sedation, demonstrating Grade 4 foveal hypoplasia (fovea plana) (1). Grade 4 foveal hypoplasia is characterized by the absence of the foveal pit, absence of outer segment lengthening, and absence of outer nuclear layer widening. Optical coherence tomography was performed using a handheld device (ENVISU C class 2300 [Leica Microsystems, Wetzlar, Germany]; <4-μm axial resolution). The acquisition protocol used a 10 × 10-mm scanning window. The 3-dimensional raster scan program for both scan sequences comprised 100 B-scans and 500 A-scans per B-scan line. The acquisition time was short (1.9 seconds) to facilitate successful image acquisition with minimal disruption of quality, thereby avoiding measurement bias. Figure 1B displays a normal fovea from a healthy, age-matched, Caucasian male control for comparison. On latest follow-up at 58 months old, the patient's binocular visual acuity (VA) was 1.2 logMAR.

**FIG. 1.**
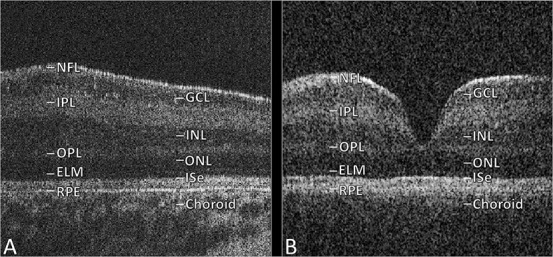
OCT images for 28-day-old with fovea plana (**A**) and 24-day-old control subject (**B**). ELM, external limiting membrane; GCL, ganglion cell layer; ISe, inner segment ellipsoid; I/OPL, inner/outer plexiform layer; I/ONL, inner/outer nuclear layer; OCT, optical coherence tomography; NFL, nerve fiber layer; RPE, retinal pigment epithelium.

Albinism is associated with nystagmus and foveal hypoplasia (1–3). Typical foveal hypoplasia can be graded on a scale of 1–4, based on morphological characteristics seen on OCT imaging, whereas atypical foveal hypoplasia is seen exclusively in achromatopsia (1). In our unit's recent study, we found that foveal hypoplasia grading was the best predictor of future VA compared with preferential looking testing, foveal developmental index (the ratio of inner retinal layers vs total foveal thickness), outer segment length, and photoreceptor length (1). Preferential looking testing was only successful in 68.1% of patients, compared with a 90% success rate for handheld OCT (1). Of note, our study found that infants with Grade 4 foveal hypoplasia had a mean predicted VA of 1.0 logMAR at school age (1). In this illustrative case, the patient's final VA fell within 2 lines of this figure.

Predicting future VA using OCT can help parents in planning adjustments to optimize their child's development and educational attainment (4). From the clinician's perspective, the high degree of foveal hypoplasia in this case likely accounts for the limitation in VA. However, if VA was worse than predicted according to foveal hypoplasia grading, then suspicion should be raised for other co-existing pathology limiting the child's VA. This could include amblyopia, uncorrected refractive error, anterior segment conditions, retinal dystrophy, or neurological disorders.

STATEMENT OF AUTHORSHIP

Category 1: a. Conception and design: S. R. Rufai, H. Lee, and I. Gottlob; b. Acquisition of data: S. R. Rufai, H. Lee, and I. Gottlob; c. Analysis and interpretation of data: S. R. Rufai, H. Lee, and I. Gottlob. Category 2: a. Drafting the manuscript: S. R. Rufai; b. Revising it for intellectual content: H. Lee and I. Gottlob. Category 3: a. Final approval of the completed manuscript: S. R. Rufai, H. Lee, and I. Gottlob.
